# Pycnodysostosis with Special Emphasis on Dentofacial Characteristics

**DOI:** 10.1155/2015/817989

**Published:** 2015-11-16

**Authors:** Aisha Khoja, Mubassar Fida, Attiya Shaikh

**Affiliations:** Section of Dentistry, Department of Surgery, The Aga Khan University Hospital, Stadium Road, P.O. Box 3500, Karachi 74800, Pakistan

## Abstract

Pycnodysostosis is an autosomal recessive disorder that manifests as osteosclerosis of the skeleton due to the defective osteoclasts mediated bone turnover. The diagnosis of this disorder is established on the basis of its characteristic features and must be differentially diagnosed with other bone disorders. Dental surgeons should be aware of the limitations and possible adverse oral complications such as osteomyelitis of bone in these patients. This will guide them in planning realistic treatment goals. This paper reports the clinical and radiographic features of pycnodysostosis with the great emphasis on its dentofacial characteristics. The aim of this case report is to give an insight into the etiology, pathogenesis, and differential diagnosis of this disorder and to prepare the dentists and maxillofacial surgeons to overcome the challenges in treating these patients.

## 1. Introduction

Pycnodysostosis is a rare genetic disorder of the bone caused by a mutation in the gene that codes the enzyme cathepsin K and has been mapped on chromosome 1q21 [1, 2]. The deficiency of this enzyme within the osteoclasts results in diminished and defective resorption of organic bone matrix and osteosclerosis of the entire skeleton [2]. The major clinical characteristics of the patients presenting with pycnodysostosis include short stature, multiple fractures of bones, craniofacial anomalies including dolichocephalic skull with patent fontanelles, frontal or occipital bossing of the head, dysplastic clavicles, unusually short distal phalanges, hypoplastic maxillary bone, and an obtuse mandibular gonial angle [3–6]. Dental features of this disorder may include retained deciduous dentition, delayed eruption of permanent dentition, crowded and malposed teeth, extremely narrow and grooved palate, anterior crossbite, lateral open bite, hypodontia, enamel hypoplasia, abnormal tooth morphology, dilacerated and hypoplastic root apices, and poor oral hygiene [3, 6].

The aim of this case report is to present the clinical and radiographic features of pycnodysostosis with special emphasis given on the associated dentofacial characteristics. Dental surgeons should be familiar of the classic features of this disorder in order to establish correct and timely diagnosis especially when they are the first to encounter these patients. In addition, they should have sound knowledge about the etiology, pathogenesis, and differential diagnosis of the disease. This will help them in proper dental management of these patients. In addition, orthognathic surgery to correct the underlying skeletal discrepancy in these patients is quite challenging and requires a thorough understanding of its limitations as well as associated risk factors which may have serious consequences if not properly addressed. Moreover, maxillary advancement with distraction osteogenesis has shown to provide optimal facial esthetics with improvement in clinical exorbitism and midface retrusion and also improve breathing and speech related problems [7]. However, the potential morbidity with any surgical intervention cannot be overlooked.

## 2. Case Report

A 13-year-and-11-month-old female from Hyderabad, Pakistan, was referred for an orthodontic treatment at our hospital with the chief complaint of “inefficient chewing function as well as old age appearance of smile with no visibility of teeth”. The patient had a marble bone disease and a history of multiple and recurrent bone fractures especially of the left midshaft tibia region which were treated conservatively. The last fracture occurred 6 months back due to fall during playing sports. On family history, elder brother also had similar condition while the parents were normal and healthy. The past medical history revealed growth hormone deficiency for which growth hormone injections were given till the age of 12 years.

## 3. Clinical and Radiographic Features

**Figure 1:**
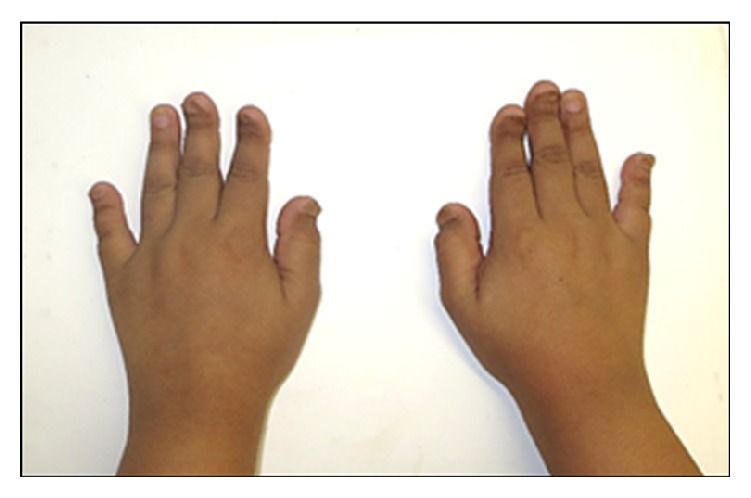
Broad hands with short, spoon shaped phalanges and dystrophic nails.

**Figure 2:**
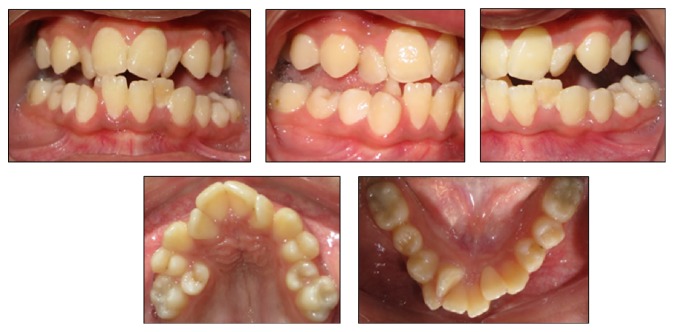
Intraoral features.

On clinical examination, the patient demonstrated with short stature with the standing height of 138 cm and weight of 58 kg. The facial form was euryprosopic with deficient midface and absence of upper incisors showing on rest and smile. The hands were broad with short, spoon shaped phalanges and dystrophic nails as shown in Figure 1. The eyes were brown-black in color with normal appearing sclera. On intraoral examination, all permanent teeth were present except for second permanent molars in both arches. There was Class III incisor relationship with reverse overjet of 2 mm, bilateral lateral open bite, narrow and grooved palate, irregular and malpositioned teeth, and v-shaped maxillary and mandibular dental arches (Figure 2). The dental hygiene was satisfactory with mild plaque and calculus deposits on upper anterior lingual surface and lower anterior labial and lingual surfaces. There was no periodontal disease; however, mild edema and color changes were observed. There were Class I carious lesions present on mandibular first permanent molars which were restored with composite fillings.

**Figure 3:**
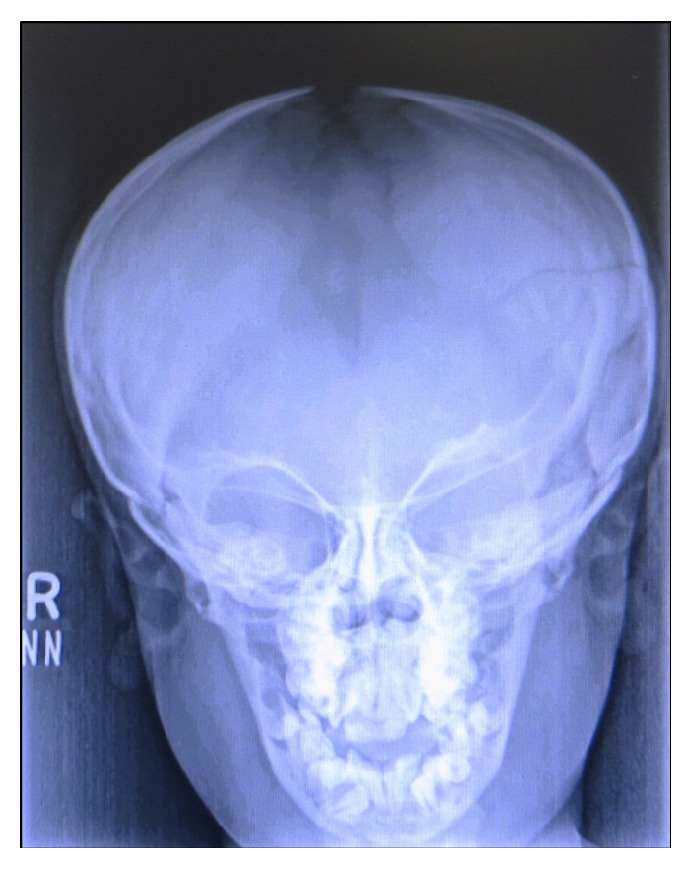
Skull radiograph with patent anterior fontanelle.

**Figure 4:**
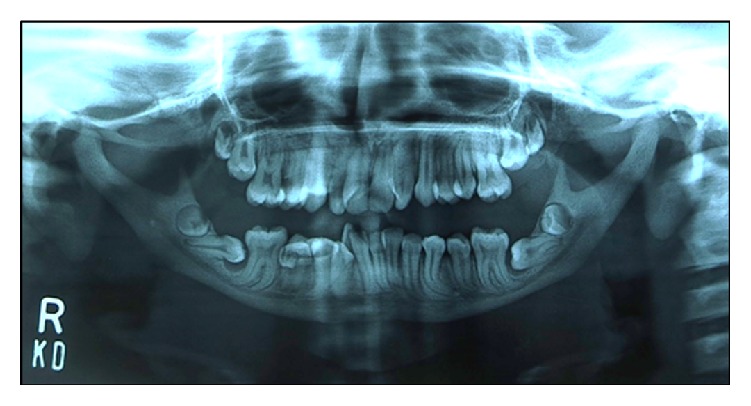
Panoramic radiographic findings.

**Figure 5:**
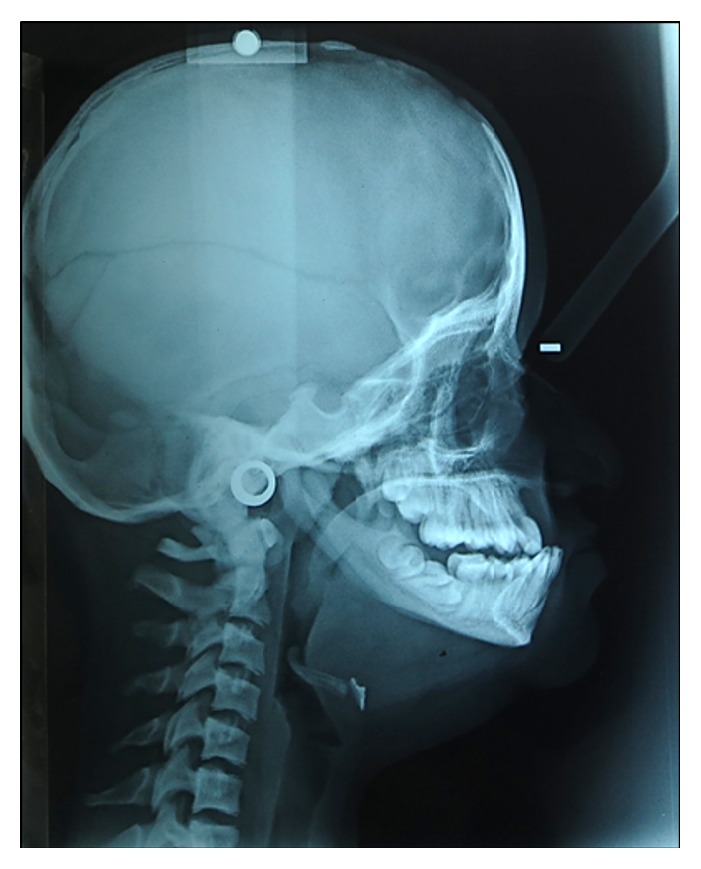
Cephalometric measurements and analysis.

The skull radiograph demonstrated frontal bossing and significant suture diastasis with patent anterior fontanelle as well as wormian bodies (Figure 3). On dental panoramic radiograph, all permanent teeth were present amongst which all second permanent molars were impacted. There were vertical and horizontal impactions of maxillary and mandibular second permanent molars, respectively. The right mandibular second molar exhibited abnormal shape and morphology. The root apices of mandibular premolars and molars showed abnormal curvature. The mandible showed a striking feature of thin and narrow body with an obtuse gonial angle and elongated condylar neck (Figure 4). The cephalometric radiographic examination showed skeletal Class III sagittal relationship of jaws (ANB = −4°), retropositioned maxilla and mandible with respect to anterior cranial base (SNA = 71°, SNB = 75°), mild tendency towards hyperdivergent pattern of growth (Go-Gn-SN = 38°), decreased anterior and posterior facial heights (N-Me = 96 mm, S-Go = 58 mm), but normal Jarabak's ratio (PFH/AFH = 60.4%). The COGS (cephalometrics for orthognathic surgery) analysis showed deficiency of mandibular body (SN = 64 mm, Go-Pog = 55 mm). The upper incisors inclination with respect to anterior cranial base appeared normal (UI-SN = 98°) whereas, lower incisors in relation with mandibular plane were proclined (IMPA = 102°). The upper lip relation with respect to Ricketts E-line appeared slightly recumbent whereas, the lower lip was normal (UL-E-line = −6 mm, LL-E-line = −1 mm). The Steiner Holdaway analysis demonstrated a retropositioned chin in relation with lower incisors (Holdaway ratio = 9 : −3). All the cephalometric measurements were compared with the standard Caucasian norms (Figure 5) [8]. On Skeletal age assessment, the patient belonged to CS-6 of cervical vertebral maturation stages as proposed by Baccetti et al. [9] with visible concavity and curvature at the lower border of second to sixth cervical vertebrae.

**Figure 6:**
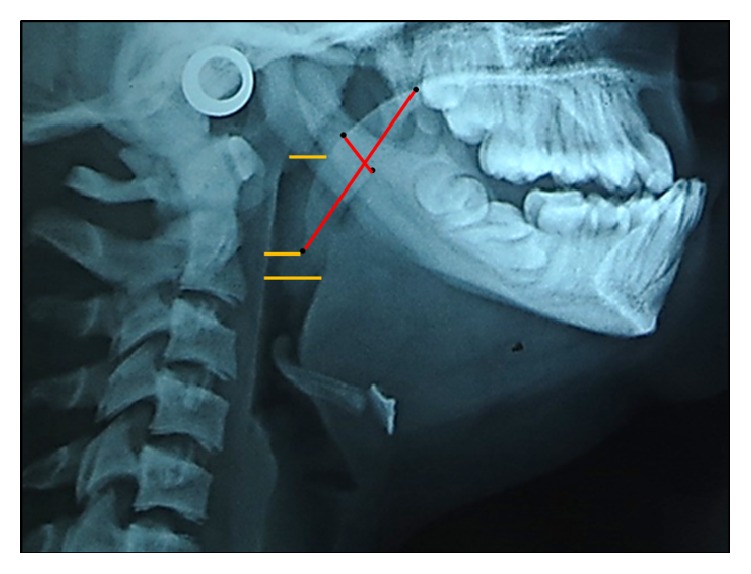
Soft palate and airway dimensions.

On airway analysis, the soft palate dimensions were 35 mm for soft palate length as measured from posterior nasal spine to tip of the soft palate (PNS-P) and 9.6 mm for soft palate thickness (maximum thickness of soft palate measured on line perpendicular to PNS-P) [10]. The upper airway space (width of airway behind middle of soft palate along parallel line to Go-B) was 8.4 mm, middle airway space (width of airway along parallel line to Go-B line through tip of the soft palate) was 7.5 mm, and inferior airway space (width of airway space along Go-B line) was 11 mm [10] (Figure 6). Despite the narrow upper airway space, there were not any problems associated with breathing during wakefulness or sleep.

## 4. Laboratory Investigations

The results for serum calcium, phosphate, and alkaline phosphatase were normal; however, the complete blood count revealed anisocytosis, hypochromic, microcytic red blood cells with the resultant iron deficiency.

## 5. Orthodontic Treatment Plan

**Figure 7:**
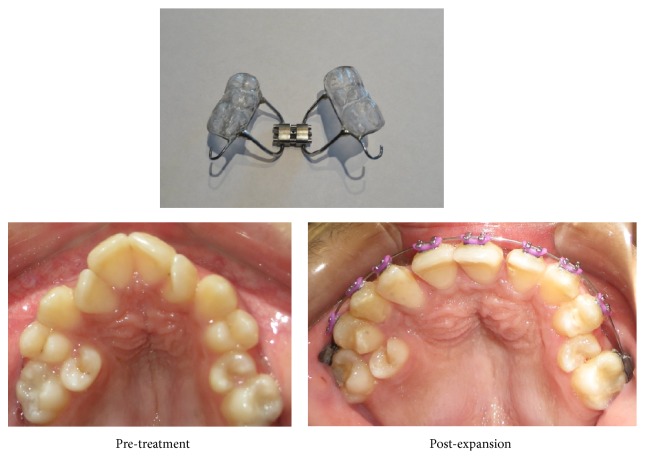
Treatment progress after maxillary orthopedic expansion.

The treatment plan decided for this patient comprised cemented upper bonded expander with the midline expansion screw which was activated 0.25 mm every alternate day using a slow expansion technique. The follow-up visits were scheduled every month to assess the overall treatment progress and radiographs were taken to see any adverse effects. Periodic scaling and polishing of teeth were carried out to maintain the oral hygiene. Meticulous attention was given on any carious lesions, white spot lesions, plaque, or calculus deposits that may arise during the course of treatment. The total duration to achieve desired orthopedic expansion was 6 months. The appliance was kept passive for the next two months to allow remodeling of tissues. Following the slow expansion technique, the next step followed was leveling and alignment of the maxillary teeth with comprehensive fixed mechanotherapy using Roth prescription 0.022 slot (Figure 7). At present, space creation for right and left maxillary second premolars is being performed using the push coil springs mechanics. Once the gross alignment of maxillary dentition is achieved, mandibular teeth will be bonded. Further treatment plan includes extractions of third molars and right mandibular lateral incisor under general anesthesia, active distalization of right upper first permanent molar, and uprighting of mandibular second permanent molars utilizing mini implant anchorage. The purpose of the entire orthodontic treatment is to achieve normal overjet, overbite, and Class I buccal occlusion and to provide optimal function and improved esthetics.

The past dental history revealed that the patient previously had extractions of few retained deciduous teeth to allow better alignment of permanent teeth. There was no postoperative infection reported and the healing of the underlying bone was normal. However, the extraction of permanent teeth is more traumatic and, therefore, requires all the possible measures to prevent osteomyelitis of bone. Hence, the extractions of the required teeth are planned to be performed by maxillofacial surgeon under general anesthesia with proper aseptic technique, minimal trauma, selective decortication and saucerization if required, and the use of intravenous prophylactic antibiotics.

## 6. Discussion

Pycnodysostosis is an inherited disorder of the bone in which impaired osteoclasts mediated bone resorption causes osteosclerosis [6, 11]. The incidence is estimated to be 1.7 per million births [11]. It was first described by Maroteaux and Lamy [5], in 1962, and is also called Toulouse-Lautrec syndrome after the French artist and painter Henri de Toulouse Lautrec, who suffered from this disorder [12, 13]. The diagnosis of this disorder is established on the basis of its characteristic features and must be differentially diagnosed with other bone disorders particularly cleidocranial dysostosis, acroosteolysis, osteogenesis imperfecta, and osteopetrosis [4, 14].

Cleidocranial dysostosis is transmitted as an autosomal dominant inheritance. The clinical features include normal stature or minor degree of dwarfism, complete or partial absence of clavicular bone, drooping shoulders, and normal texture of bones except for increased density of the base of the skull [15]. In contrast, pycnodysostosis is autosomal recessive disorder and the clavicular bone is always present and is rarely affected. Acroosteolysis is manifested as characteristic face with prominent eyes and upturned nose, some degree of hypertelorism, stunted body stature, absence of frontal sinuses, trunk deformities, and shortening of the phalanges. However, the absence of the obtuse mandibular angle, which is pathognomonic of pycnodysostosis, does not occur in acroosteolysis [16]. In osteogenesis imperfecta, there is an occurrence of more severe multiple bone fractures as compared to pycnodysostosis. In addition, associated features such as choanal atresia and blue sclera are almost always present [14]. Osteopetrosis is presented with a generalized increased in bone density [14]. The associated features include splenomegaly, hepatomegaly, lymphadenopathy, and jaundice. In malignant form, severe aplastic anemia can occur due the obliteration of the medullary spaces and is usually fatal at an early age [17]. Unlike osteopetrosis, anemia and hepatosplenomegaly if present in patients with pycnodysostosis do not occur due to the obliteration of medullary spaces but are due to the presence of active hematopoiesis [17].

Once a proper diagnosis is made, patients with pycnodysostosis can have a normal life expectancy [18]. However, dental treatment in them requires vigilant care to prevent serious consequences or complications [4]. Osteomyelitis is the most serious complication that may occur due to any local condition which interferes with the blood supply of the bone causing tissue necrosis and infection [19]. With an advancing age, these patients are at increased risk of developing osteomyelitis of jaws following tooth extractions due to poor bone healing. In addition, due to the defective osteoclastic activity, the resultant bone formation gradually jeopardizes the vascular supply by slowly eliminating the medullary spaces [20]. Therefore, the extractions of the required teeth for orthodontic alignment in the abovementioned case report should not be delayed. In Japanese literature, out of fifty-four case reports by Muto et al. [21], nine cases developed osteomyelitis with an age of twenty years or more. In addition, dental surgeons should also be aware of the possibility of causing mandibular fracture following tooth extraction due to the increased bone density [22]. Vigilant and strict care is required which includes avoiding traumatic extractions, utilizing proper aseptic techniques, and use of antibiotic prophylaxis especially in the adults. The patients should be instructed to maintain excellent oral hygiene and are encouraged for regular dental checkups. Periodic scaling of teeth as well as restorations of carious lesions should be carried out in order to prevent the risk of developing osteomyelitis associated with the bacterial pathogens [4].

In these patients, malaligned and irregular dentition may pose difficulty to maintain good oral hygiene [6]. Some authors recommend early orthodontic treatment to relieve dental crowding [23]. However, the second phase of comprehensive treatment will be required in permanent dentition phase. Therefore, early treatment with multibanded fixed appliances will prolong the overall treatment duration as well as increasing the chances of developing white spot lesions. Others suggest planned and sequential extractions in early age to guide the eruption of permanent teeth in better position [4, 24]. In addition, early extractions of retained deciduous teeth may reduce the chances of impaction of the permanent teeth. Ortegosa et al. [24] in their study planned serial extractions in a patient with pycnodysostosis, aged 6 years and 9 months to reduce crowding and for correction of abnormal eruption sequence. In addition, preventive dental education programs and strict oral hygiene instructions should be instituted to them right from an early age [18].

In the present case, maxillary orthopedic expansion with bonded expander was performed with successful results. According to Ortegosa et al. [24], activation of midline screw exerts force upon the teeth and the palate resulting in the separation of the maxillary and palatine process in the median palatal suture region. However, the osteosclerosis of bone in these patients may increase the risk of nonrupture of the median palatine suture, fracture of the alveolar rims, mobility of the teeth, and maxillary osteomyelitis. In contrast, we performed treatment with bonded expander using slow expansion protocol with 0.25 mm of activation twice a week with no signs of pain, edema, or swelling. In addition, there were no associated symptoms of tooth mobility, root resorption, or maxillary osteomyelitis. However, the evidence for the long term retention of these protocols in pycnodysostosis has not yet been reported. Therefore, clinical trials should be performed regarding the success and stability of orthopedic expansion, maxillary protraction with facemask, and orthodontic fixed appliances.

Orthognathic surgeries to correct the severe skeletal discrepancy can be considered in these patients [12, 25]. Hernández-Alfaro et al. [12], in their case report of patient with pycnodysostosis, described bimaxillary orthognathic surgeries using rigid fixation and bone grafts for treating dentofacial deformities. The results obtained were esthetically and functionally stable. However, there is an increased risk of osteomyelitis and nonunion or malunion of bony ends in these patients, and hence this represents a challenge for the maxillofacial surgeon. In addition, other complications with conventional orthognathic surgery may include infection due to presence of bone graft and fixation with bone plates and screws, transection of bilateral inferior alveolar nerves, and velopharyngeal problems [7]. Extraoral distraction osteogenesis could minimize the risk of infection, improve breathing in patients with obstructive sleep apnea, and promote the desired movement without jeopardizing speech function [7]. Successful advancement of maxilla by distraction osteogenesis with rigid external distractor device has been reported in many case reports [26–28]. Nørholt et al. [28] performed distraction osteogenesis of maxilla in a 15-year-old girl with pycnodysostosis and reported stable bony consolidation after 13 months of Le Fort I osteotomy followed by 6 weeks of external distraction. Subcranial Le Fort III advancement with internal distraction osteogenesis was performed in another case report of a 13-year-old girl with pycnodysostosis and obstructive sleep apnea disorder [7]. It has been observed that this procedure not only improved the symptoms associated with obstructive sleep apnea but also resolved her exorbitism with significant improvement of facial appearance. However, the success of such invasive procedures depends on skills of the surgeon, aseptic protocols, length and duration of surgery, and individual bone physiological response. Therefore, the risk benefit ratio should always be weighted before undergoing such invasive procedures in these patients.

## 7. Conclusions

Patients with pycnodysostosis should be carefully examined for their clinical and radiographic features in order to establish correct diagnosis. Meticulous attention should be paid in handling them due to their fragile bone resulting from defects in osteoclasts function. Special emphasis should be given in maintaining good oral hygiene and preventive measures must be maintained on frequent dental visits. Orthopedic expansion using slow expansion protocol and orthodontic alignment of teeth can be carried out in these patients with success. However, the outcome of orthodontic treatment may vary on individual basis. Therefore, further in vivo and in vitro studies in animals and humans should be carried out to see the impact of orthodontic treatment on osteoclasts, bone metabolism, and healing of tissues in these individuals. In addition, these patients should always be informed about the possible risks and complications before undergoing any surgical procedure.
